# Ipsilateral pubic ramus fracture during total hip arthroplasty is not rare: does it matter?

**DOI:** 10.1007/s00402-024-05368-5

**Published:** 2024-05-14

**Authors:** Young-Seung Ko, Han Jin Lee, Hong Seok Kim, Jeong Joon Yoo

**Affiliations:** 1 Department of Orthopaedic Surgery, Hallym university Dong-Tan Sacred Heart Hospital, Hwaseong, South Korea; 2 Department of Orthopaedic Surgery, Hanil Hospital, Seoul, South Korea; 3 Department of Orthopaedic Surgery, Seoul National University College of Medicine, 101, Daehak-ro, Jongno-gu, Seoul, South Korea

**Keywords:** Total hip arthroplasty, Periprosthetic fractures, Ramus fracture, Rami fracture

## Abstract

**Introduction:**

Periprosthetic fractures in total hip arthroplasty (THA) have been well described and studied. However, there is a lack of reports on ipsilateral pubic ramus fractures during THA due to the rare occurrence of such fractures and ambiguity of symptoms. With the use of postoperative computed tomography (CT) examinations, we have identified that asymptomatic ipsilateral pubic ramus fractures occur frequently during THA. This study aims to evaluate the incidence, location, clinical outcomes, and risk factors of ipsilateral pubic ramus fractures during THA.

**Methods:**

From May 2022 to March 2023, a single surgeon performed 203 THAs in 183 patients at a single institution. All patients underwent postoperative CT scans three days after THA. The patients with ipsilateral pubic ramus fractures were followed up for a minimum of six months. Basic demographics, osteoporosis, general conditions of the operations, and outcomes of THA were investigated in all patients.

**Results:**

Twenty-two cases (10.8%) of ipsilateral pubic ramus fractures were identified on postoperative CT scans. All fractures were located near the origin of the superior or inferior pubic ramus. Five fractures were detected on simple postoperative radiographs. The fractures did not cause any further complications at a minimum of six-month postoperative follow-up. Univariate and multivariate analyses did not identify any risk factors associated with these fractures.

**Conclusions:**

Although the incidence of ipsilateral pubic ramus fractures during THA is high, treatment is not required as they do not cause any significant clinical symptoms or affect the prognosis of THA. However, the possibility of occurrence of these fractures must be explained to the patients before surgery.

**Supplementary Information:**

The online version contains supplementary material available at 10.1007/s00402-024-05368-5.

## Introduction

Currently, cementless acetabular components are commonly employed in total hip arthroplasty (THA) procedures, utilizing the under-reaming technique and press-fit impaction [23]. Press-fit impaction of the acetabular component with or without screw fixation has become the most widely used fixation technique [8]. However, during impaction, a periprosthetic pelvic fracture can occur. Fortunately, it has been rarely reported [9]. Simple radiographs alone may not be sufficient to detect a periprosthetic fracture of the pelvis immediately after its occurrence; other modalities, such as computed tomography (CT) or magnetic resonance imaging (MRI), are usually required to confirm the fracture.

As part of another study protocol that obtained postoperative CT images in consecutive primary THAs, we identified postoperative ramus fractures and found that the incidence of these fractures was unexpectedly high.

Previously, the following risk factors for perioperative periprosthetic fractures around the acetabulum were studied: under-reaming or oversizing of the cup, osteoporosis, and a preoperative diagnosis of rheumatoid arthritis [2, 5, 6, 12, 15, 16, 26]. However, little is known about the incidence, risk factors, and outcomes following periprosthetic ramus occult fractures.

Therefore, we aimed to assess (1) the prevalence of occult fractures of the ipsilateral ramus during primary THA, (2) location of these fractures, (3) radiologic and clinical outcomes, and (4) risk factors contributing to these fractures.

## Methods

This was a case series study with retrospective review of the medical records upon approval of the Institutional Review Board of our hospital (IRB no.: H-2302-040-1402). The requirement for obtaining informed consent was waived due to the retrospective nature of the study.

### Participants

Between May 2022 and March 2023, all 189 patients undergoing 209 primary cementless THAs at our institution were included in the study and they underwent CT scans three days after the index surgery. THAs performed during this time were not excluded from this imaging protocol. Exclusion criteria for patients in this study were prior history of pelvic osteotomy, pelvic trauma, previous joint infection, and usage of the automated surgical impactor. Six patients, in whom an automated surgical impactor was used, were excluded in order to control for variables and reduce bias. A total of 183 individuals with 203 hips were included.

A total of 183 participants (126 females and 57 males), with an average age of 55.5 ± 15.0 years (range, 16–83 years) participated in this study. Their average body mass index (BMI) was 25.4 ± 4.2 Kg/m^2^ (range, 17.7–37.4 kg/m^2^).

Preoperative diagnoses included osteonecrosis of the femoral head in 96 hips, primary osteoarthritis or arthritis due to dysplastic hip in 84 hips, sequelae of Perthes’ disease in 12 hips, inflammatory arthritis in 8 hips, and other in 3 hips (Table 1).

Dual-energy X-ray absorptiometry (DXA) was routinely performed in individuals over the age of 50 years.

**Table 1:** Demographics

	Non-fracture (n = 181 hips)	Fracture (n = 22 hips)	*p*-value
Age	55.3 ± 15.1	57.5 ± 14.5	0.516
Gender			
Female Male	112 (61.9%) 69 (38.1%)	14 (63.6%) 8 (36.4%)	0.873
Laterality			0.872
Right	102 (56.4%)	12 (54.5%)	
Left	79 (43.6%)	10 (45.5%)	
BMI (kg/m^2^)	25.5 ± 4.3	24.2 ± 3.5	0.165
Diagnosis			0.577
Osteonecrosis of the femoral head	85 (43.6%)	11 (50.0%)	
Primary arthritis or arthritis due to hip dysplasia	77 (40.8%)	7 (31.8%)	
Sequelae of Perthes disease	8 (4.4%)	4 (18.2%)	
Inflammatory arthritis (AS, RA, or JRA)	8 (4.4%)	0 (0%)	
Other	3 (1.7%)	0 (0%)	
Charlson Comorbidity index	2.08 ± 1.6	2.00 ± 1.6	0.827
ASA score	2.09 ± 0.5	2.00 ± 0.3	0.242
Osteoporosis	35/146 (24.0%)	6/19 (31.6%)	0.470

*Abbreviations* BMI, body mass index; AS, ankylosing spondylitis; RA, rheumatoid arthritis; JRA, juvenile rheumatoid arthritis; ASA, American Society of Anesthesiologists

### Operation

A single hip arthroplasty surgeon (JJY) with more than 20 years of experience in a tertiary referral hospital performed all index operations. The surgical procedure for THA was carried out in the lateral decubitus position using the modified direct lateral approach [20]. The patient secured to the table using the lateral hip positioner (Online resource 1) [19]. True hemispheric acetabular component designs were used, such the Bencox Mirabo cup (Corentec, Cheonan, Korea) and G7^®^ (Zimmer Biomet, Warsaw, IN, USA). After under-reaming the acetabulum by 1 mm, the hemispheric cups were inserted using the press-fit technique.

Impaction of the acetabular component was performed two or three times with a surgical mallet (1.8 kg) swung by gripping its neck. Depending on the rigidity of the press-fit, two dome screws or no dome screws were inserted.

The angle of the acetabular cup was set to achieve an inclination of 30–45° and an anteversion of 15–25° [3].

We performed routine total hip arthroplasty without any additional procedures, such as internal fixation of fractures, for all participants.

### Postoperative CT scan

**Fig. 1:**
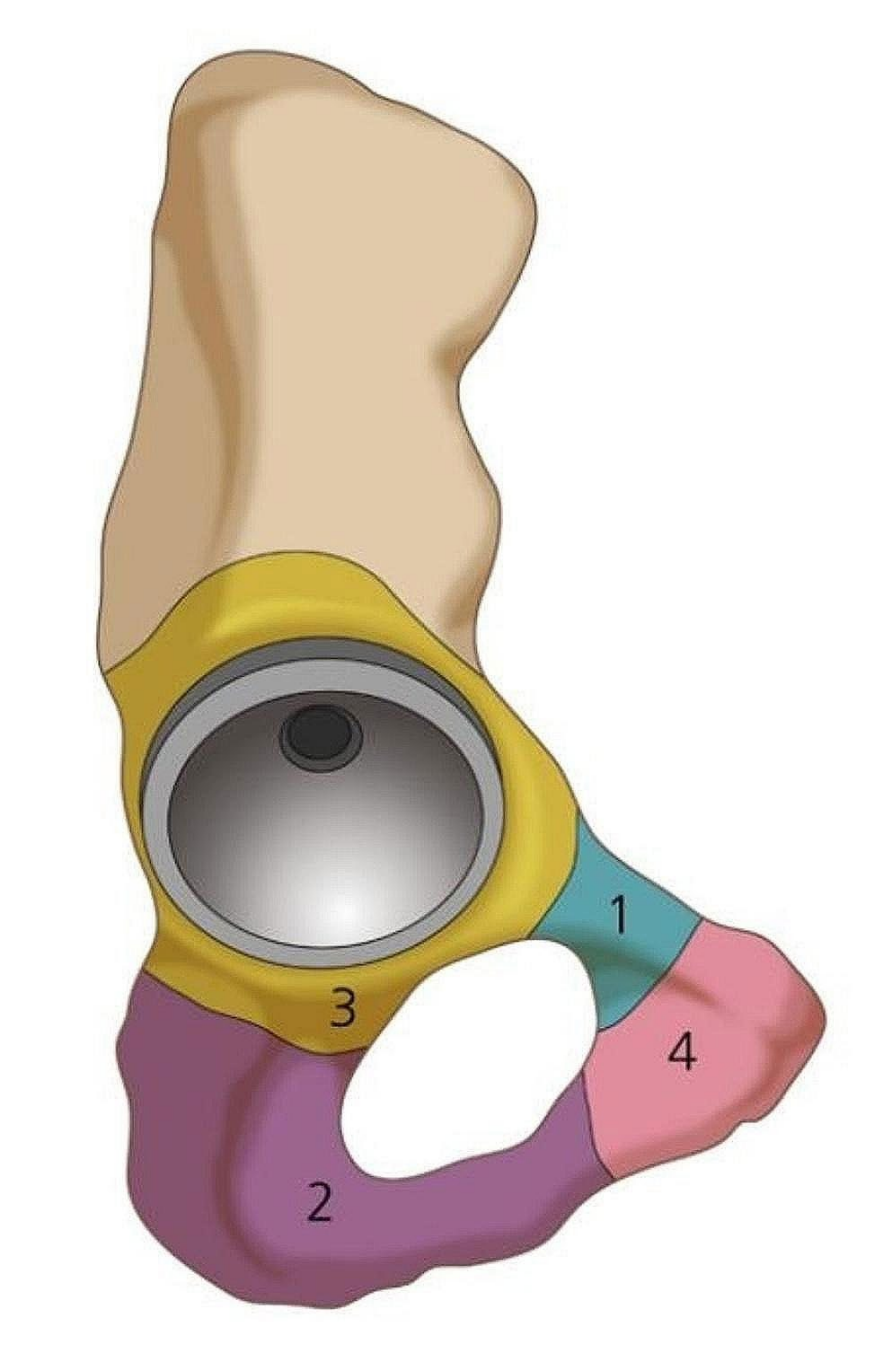
Location of fractures; (**1**) Superior pubic ramus, (**2**) Inferior pubic ramus, (**3**) Periacetabulum and (**4**) Pubic symphyseal area

We differentiated occult fractures from periprosthetic fractures that were noticed during surgery, and we defined occult fractures as those that were not seen on either intraoperative findings or intraoperative radiographs but could only be checked on the postoperative 3D CT images of the pelvis. Based on where the fractures had occurred, we divided the location of fractures into the following five groups: superior pubic ramus, inferior pubic ramus, both rami, the periacetabulum, and pubic symphyseal area (Fig. 1). We identified the periacetabulum-to-rami boundary as the lateral margin of the obturator foramen. The pubic symphyseal region and rami were defined as being separated by the medial edge.

### Functional recovery

Regardless of the fracture, partial weight-bearing with a crutch gait or a walker was recommended for the first 6 weeks after surgery. Subsequently, full weight-bearing was allowed as tolerated. Patients were followed up for 6 weeks, 6 months, 12 months, and then annually after the surgery.

### Radiologic outcome and functional outcome

Radiographic evaluations were performed by two independent fellowship-trained orthopedic surgeons who did not participate in the initial arthroplasties (HSK and YSK). In addition to the postoperative CT scans, anteroposterior and cross-table lateral radiographs were obtained at each follow-up, along with the inlet, outlet, and oblique views of the pelvis. Acetabular components were considered loose if there was a change in the alignment by 4° or more or 4 mm [13].

Clinical evaluation was conducted using the modified Harris Hip score (worst score 0, best score 100) [4].

### Statistical analysis

Statistical analysis in this study involved comparing continuous variables using the t-test or Mann-Whitney U test and comparing dichotomous variables using the chi-square test or Fisher exact test. Additionally, multivariate logistic regression analysis was used. A significance level of *p* < 0.05 was used to determine statistical significance. Statistical analyses were performed using IBM SPSS Statistics for Windows, version 25.0 (IBM Corp., Armonk, NY, USA).

## Results

### Incidence of fractures

Periprosthetic fractures of the acetabulum were not detected during surgery. In postoperative CT scans, the ipsilateral ramus fracture was found in 22 hips (10.8%). All contralateral rami were intact. Before identifying ramus fractures that occurred during THA, there were 12 cases of ramus fractures among 90 THAs. Following awareness of this issue and formulation of this study, 10 fractures were identified in 113 hips. Despite being aware of the possibility of occurrence of ramus fracture, there was no statistically significant difference in the fracture incidence.

### Location of fractures

**Fig. 2:**
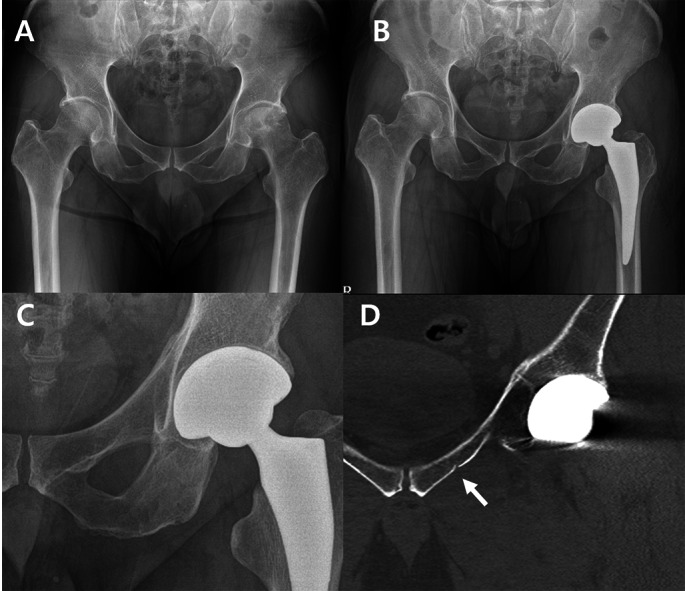
Radiographs showing superior pubic ramus fracture during total hip arthroplasty. (**A**) Preoperative anteroposterior view. (**B**) No fracture lines are visible on the postoperative hip anteroposterior radiograph. (**C**) Magnified view. (**D**) The postoperative hip computed tomography coronal view shows incomplete fracture lines (arrow) on the left superior pubic ramus

**Fig. 3:**
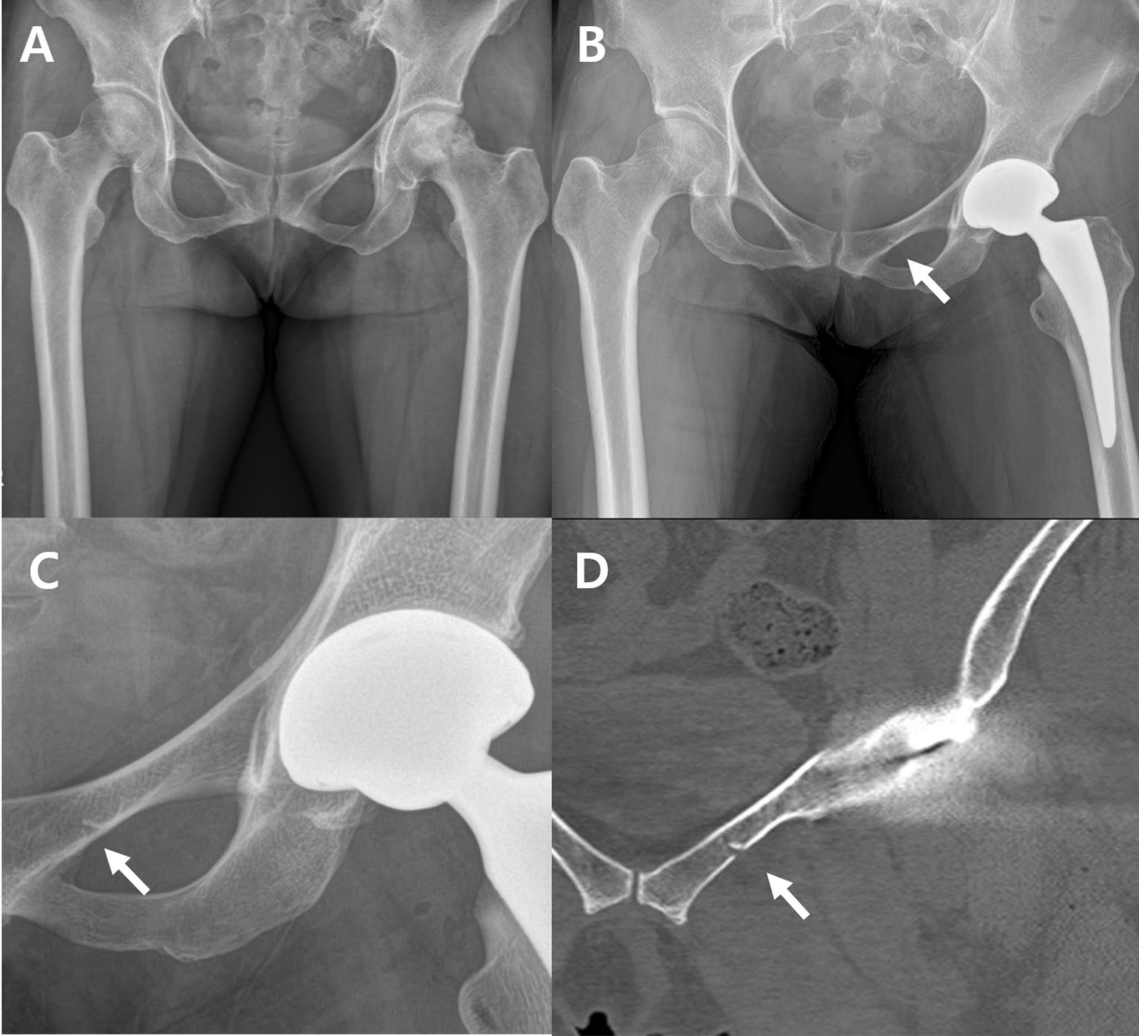
Radiographs showing superior pubic ramus fracture during total hip arthroplasty. (**A**) Preoperative anteroposterior view. (**B**) incomplete fracture lines on the left superior pubic ramus are visible on the postoperative hip anteroposterior radiograph. (**C**) Magnified view. (**D**) The postoperative hip computed tomography coronal view shows an incomplete fracture line on the left superior pubic ramus

**Fig. 4:**
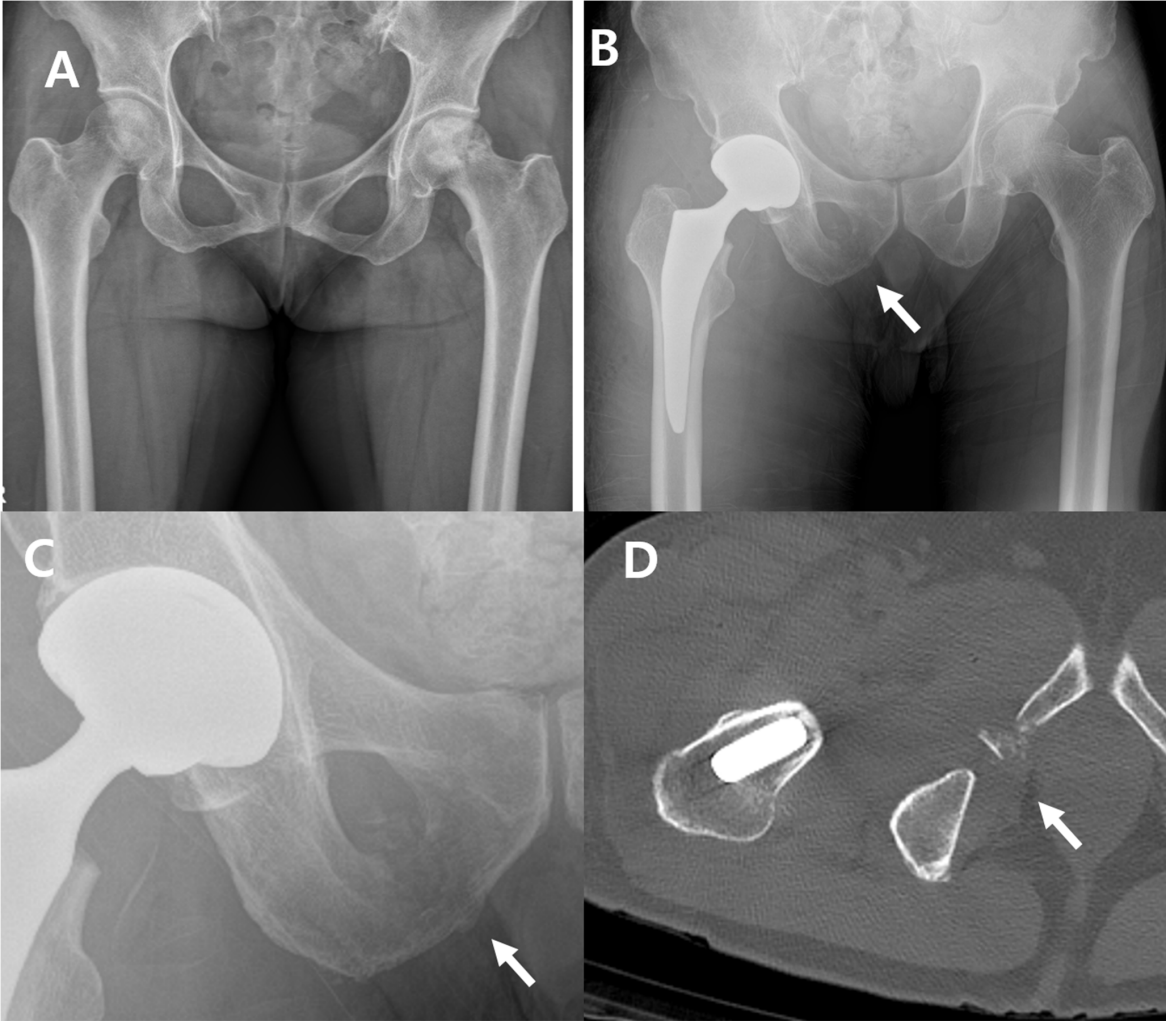
Radiographs showing an inferior pubic ramus fracture during total hip arthroplasty. (**A**) Preoperative anteroposterior view. (**B**) Fragmented fractures (arrow) on the right inferior pubic ramus are visible on the postoperative hip anteroposterior radiograph. (**C**) Magnified view. (**D**) The postoperative hip computed tomography axial view reveals comminuted fractures on the right inferior pubic ramus

Occult fractures were most frequently found in the superior pubic ramus, accounting for 10 out of 22 hips (45.4%) (Figs. 2 and 3). Fractures of the inferior pubic ramus were observed in 9 hips (40.9%) (Fig. 4), while 1 hip (4.5%) exhibited fractures in both pubic rami. Fractures involving the peri-acetabulum were observed in 2 hips (9.0%), with one hip showing an extension of the fracture into the superior pubic ramus and the other hip showing extension of the fracture into the inferior pubic ramus. No fractures were observed in the pubic symphyseal area (Table 1).

### Radiological outcome and clinical outcome

In hips with ramus fractures, the average cup inclination was 34.3°± 4.0° and anteversion was 26.3°±11.3°. On the other hand, cup inclination was 33.9°±4.8° and anteversion was 25.1°±9.5° on average in hips without any fractures with a p-value of 0.775 and 0.590, respectively.

All acetabular cups demonstrated stability with no discernible evidence of cup migration or loosening. Simple radiographs obtained after six months from the index surgery showed no evidence of further displacement or apparent fracture line.

The mean preoperative modified Harris hip score of 51.8 points (range, 47-84 points) improved to 94.6 points (range, 85-100 points) at the time of the postoperative 6-month follow-up in the non-fracture group: 53.1 points (range, 45-70 points) to 93.7 points (range, 82-100 points). Throughout the entire follow-up period, including the immediate postoperative period and up to 6 weeks postoperatively, none of the patients reported abnormal inguinal pain or tenderness. None of the patients required walking support after 6 weeks postoperatively.

### Risk factors

After controlling for potentially relevant confounding variables, including sex, age, BMI, size of each cup, cup position, cup design, manufacturer of the implants, or presence of osteoporosis, no identifiable risk factors were found to be associated with an increased risk of occult periprosthetic fracture in the multivariate logistic regression analysis.

## Discussion

Periprosthetic acetabular fracture during primary THA is a rare but reported complication. To the best of the author’s knowledge, this is the first report investigating the prevalence and outcomes of ramus fracture during THA. We observed pubic ramus fractures in 22 of 203 primary hips (10.8%) and the fracture occurred on the superior and inferior pubic rami and periacetabular area. None of the patients reported additional pain or tenderness during the entire follow-up period, and all patients achieved bony union. Risk factors associated with periprosthetic rami fractures were not identified.

**Fig. 5:**
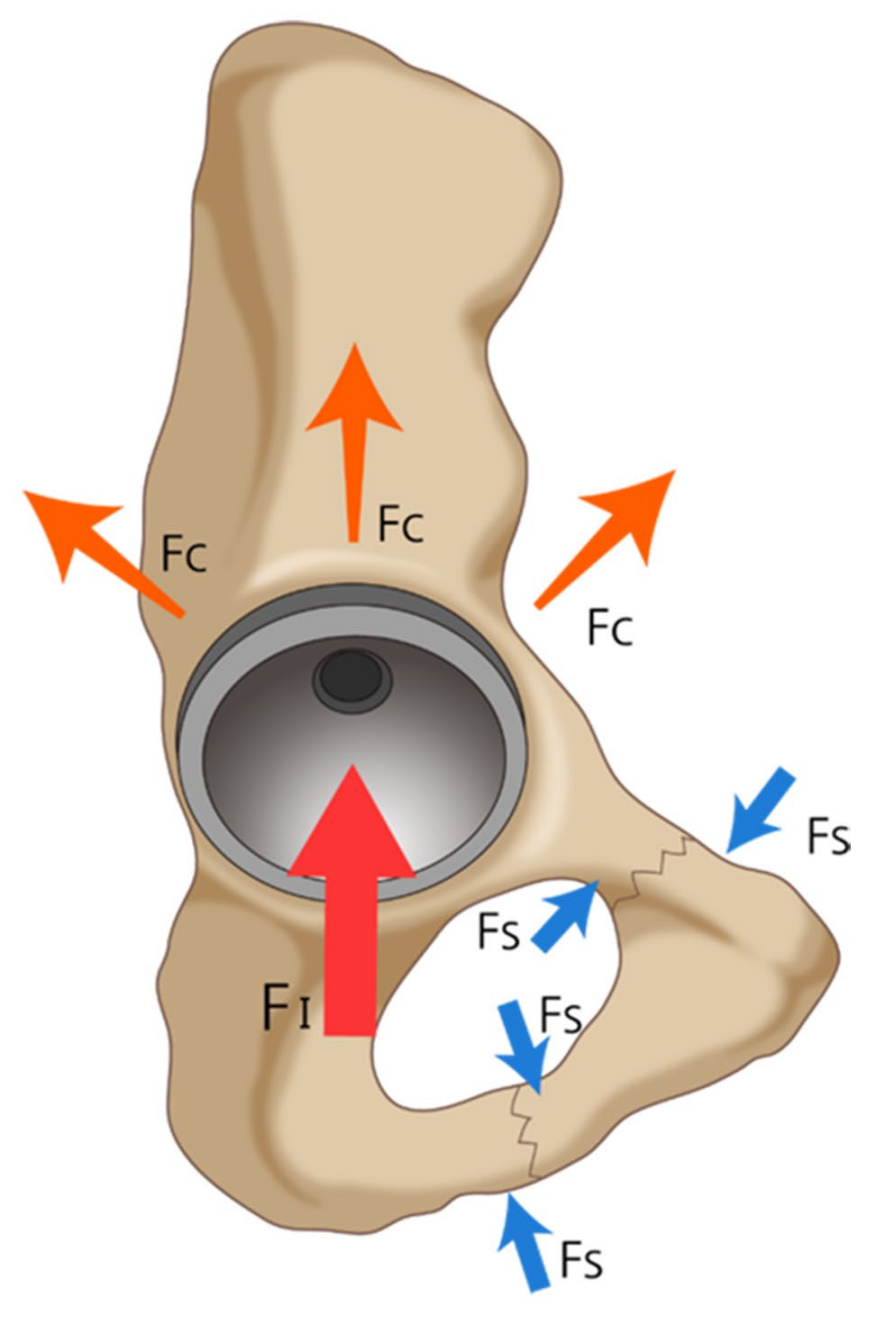
Force distribution during cup impaction. The impulsive force of cup impaction in the superior, medial, and posterior directions transforms into compressive and shear stresses on the rami. FI, impaction force; FC, compression force; FS, shear force

Femoral fractures have been extensively studied and documented [1, 10, 25]; however, there is a dearth of reports and studies on periprosthetic acetabular fractures. It is challenging to detect fractures of the ramus and acetabulum during THA on plain radiographs due to the complicated morphology of the pelvic bone, its cancellous nature, and broad soft tissue coverage. Hasegawa et al. studied periprosthetic occult fractures around the acetabulum after primary THA and reported 8.4% of occult fractures using perioperative CT scans [11]. In our study, we utilized postoperative CT scans to detect not only occult periacetabular fractures but also fractures in the rami. Fractures in rami were more prevalent than those in the periacetabular area. We postulated that the impulsive force of cup impaction in the superior, medial, and posterior directions transforms into compressive and shear stresses on the rami, which lead to fractures at the weak point of the rami (Fig. 5). The rate of ipsilateral ramus fracture was unexpectedly high. Although surgeries were only conducted by an experienced surgeon who performed more than 200 THA procedures per year, a significant prevalence was identified. There was no statistically significant difference in the prevalence of surgery before and after identifying and attending to the fracture.

In our study, all patients achieved bone ingrowth fixation, and no additional surgical interventions were necessary during the follow-up. All patients were able to ambulate without the assistance of a walking aid. However, it should be noted that postoperative pain following THA may mask any bony pain associated with the periprosthetic ramus fracture. Nonetheless, routine 6-week protective weight-bearing measures and postoperative pain management may be sufficient for adequate bony union of these fractures. Hasegawa et al. have similarly reported that periprosthetic occult fractures of the acetabulum did not require further intervention [11]. Thus, even in the event of perioperative detection of a ramus fracture, patients can be reassured that further intervention may not be necessary [7, 24]. Even if these fractures do not require any special care, the surgeon should be aware that they can occur during THA. Medical disputes or lawsuits may arise if surgeons fail to adequately inform their patients. Before the operation, the surgeon must inform the patient that ramus fractures might occur and educate them that additional care is seldom needed.

On the other hand, delayed periacetabular fractures require a different approach as they result from osteolysis around the acetabular component, which may take some time to develop after primary surgery [21]. Stress fractures of the pubic ramus have been reported in the literature as a complication following THA, which could be successfully managed with protective weight bearing. However, acute fractures due to a fall may require more attention. Radha et al. reported a case of a pubic ramus fracture that occurred seven months after index surgery, leading to instability of the acetabular component. In this case, the instability of the well-fixed acetabular component occurred due to the fact that the pubic ramus fracture extended to the acetabulum, which was not initially detected [21]. However, occult fractures during primary THA did not affect the fixation of the cup in short term observation.

Our findings indicate that bone quality, evaluated by dual-energy X-ray absorptiometry, was not correlated with the occurrence of intraoperative ramus fractures. Osteoporosis was diagnosed in 31.6% of the hips in the fracture group, while 24.0% of the hips in the non-fracture group were diagnosed with osteoporosis. The association between osteoporosis and intraoperative fracture is controversial. Some studies have suggested that sclerotic unyielding under-reamed acetabulum with good bone quality may be a reason for the fracture [22], while others have mentioned that osteoporosis may be a predisposing factor [11]. In our study, no risk factor was identified in the multivariate logistic regression analysis.

The design of the acetabular component may influence the prevalence of occult ramus fractures. Hasegawa et al. reported that among the other possible risk factors, only the use of a peripheral self-locking cup was associated with an increased risk of fractures [11]. In our study, only a hemispherical cup was used. The incidence of fractures may increase when the other types of designs are utilized. The size and position of the acetabular component were not associated with an increased risk of fractures.

We hypothesized that the patient’s position, either lateral decubitus or supine, and the surgical approach, anterolateral, direct lateral, or posterolateral, may affect the occurrence of ramus fracture. Due to the rarity of this fracture, none of the studies have shown an association between the patient’s position and occurrence of the fracture. Most studies reporting on periprosthetic intraoperative fractures were performed using the posterolateral approach with the patient in the lateral decubitus position [11, 14]. In our study, all index surgeries were performed using the direct lateral approach with the patient in the lateral decubitus position. We assumed that the prevalence of ramus fractures would differ among other surgical approaches, such as direct anterior approach with the patient in the supine position due the counter effect of the operating bed against the impaction force [18]. A future systematic review or meta-analysis is warranted.

Moreover, the influence of the surgeon’s impaction force on the prevalence of ramus fractures was also acknowledged in our study. The minimum impaction force required for a press-fit of an acetabular component in primary THA can vary based on several factors, including the size and design of the implant, the quality and thickness of the bone, and the surgical technique used [17]. A sufficient but not overwhelming impaction force should be applied to achieve stable fixation of the implant without causing any damage to the bone or deformation of the implant. It may be possible to avoid iatrogenic fractures by using an automated surgical impactor, but further research is necessary to pinpoint its potential applications.

We acknowledge several limitations of our study. First, this study was a retrospective review, although patients were enrolled longitudinally. Future large-scale, prospective, multi-center cohorts are needed to confirm our findings. Second, our study was conducted in an East Asian country, therefore the proportion of diagnoses requiring total hip arthroplasty may differ from that in Western countries. Additionallythe mean BMI was 25.4 ± 4.2 Kg/m^2^. Although there was no significant difference in the proportion of diagnosises, the height or BMI between the two groups, our findings could not be generalized to Western countries. Third, there could be the potential of selection bias since all the index surgeries were performed by a single surgeon. However, the surgeon was fellowship-trained hip arthroplasty surgeon who conducted more than 300 total hip arthroplasty procedures annually in tertiary referral hospital.Lastly, the hip positioner might affect the fracture incidence. Mittal et al. reported that there was difference in pelvic stability among different hip positioners. Though this study provides laboratory data, its relevance to actual clinical practice remains uncertain. Nevertheless, the hip positioner utilized in this study is a commonly employed standard positioner, thus it can be regarded as representative of typical condition.

## Conclusion

The incidence in this study of ipsilateral ramus fracture during THA was 10.8%. Notwithstanding the fact that the incidence rate was higher than expected, such fractures generally do not cause significant clinical symptoms. Therefore, treatment is not required. Surgeons should be aware of the high occurrence of these fractures and should educate and reassure their patients about the risk of these fractures prior to surgery.

## Electronic supplementary material

Below is the link to the electronic supplementary material.

Supplementary Material 1

Supplementary Material 2

Supplementary Material 3

Supplementary Material 4

Supplementary Material 5
