# Correction: Ipsilateral pubic ramus fracture during total hip arthroplasty is not rare: does it matter?

**DOI:** 10.1007/s00402-024-05451-x

**Published:** 2024-09-26

**Authors:** Young-Seung Ko, Han Jin Lee, Hong Seok Kim, Jeong Joon Yoo

**Affiliations:** 1 Department of Orthopaedic Surgery, Seoul National University College of Medicine, Seoul, Korea; 2 Department of Orthopaedic Surgery, Hallym university Dong-Tan Sacred Heart Hospital, Hwaseong, Korea; 3 Department of Orthopaedic Surgery, Hanil Hospital, Seoul, Korea

**Correction to: Archives of Orthopaedic and Trauma Surgery (2024) 144:2849–2857**

10.1007/s00402-024-05368-5

In the original version of this article, affiliation details for all author were incorrect. Affiliation details should have been:

Young-Seung Ko

^1^Department of Orthopaedic Surgery, Seoul National University College of Medicine, Seoul, Korea 

^2^Department of Orthopaedic Surgery, Hallym university Dong-Tan Sacred Heart Hospital, Hwaseong, South Korea 

Han Jin Lee

^1^Department of Orthopaedic Surgery, Seoul National University College of Medicine, Seoul, Korea 

^3^Department of Orthopaedic Surgery, Hanil Hospital, Seoul, Korea

Hong Seok Kim

^1^Department of Orthopaedic Surgery, Seoul National University College of Medicine, Seoul, Korea

Jeong Joon Yoo

^1^Department of Orthopaedic Surgery, Seoul National University College of Medicine, Seoul, Korea

