# Supplementary material for: Ipsilateral pubic ramus fracture during total hip arthroplasty is not rare: does it matter?
Source: Arch Orthop Trauma Surg. 2024 May 14;144(6):2849–57. doi: 10.1007/s00402-024-05368-5 (PMC11211179; doi:10.1007/s00402-024-05368-5)
Supplement: Supplementary file 5 — Supplementary Material 5 [file 402_2024_5368_MOESM5_ESM.pdf]

Ipsilateral pubic ramus fracture during total hip arthroplasty is not rare: Does it matter?

Young-Seung Ko[1] MD; Han Jin Lee[2] MD; Hong Seok Kim[1] MD, PhD; Jeong Joon Yoo[1] MD, PhD

Electronic Supplementary Material

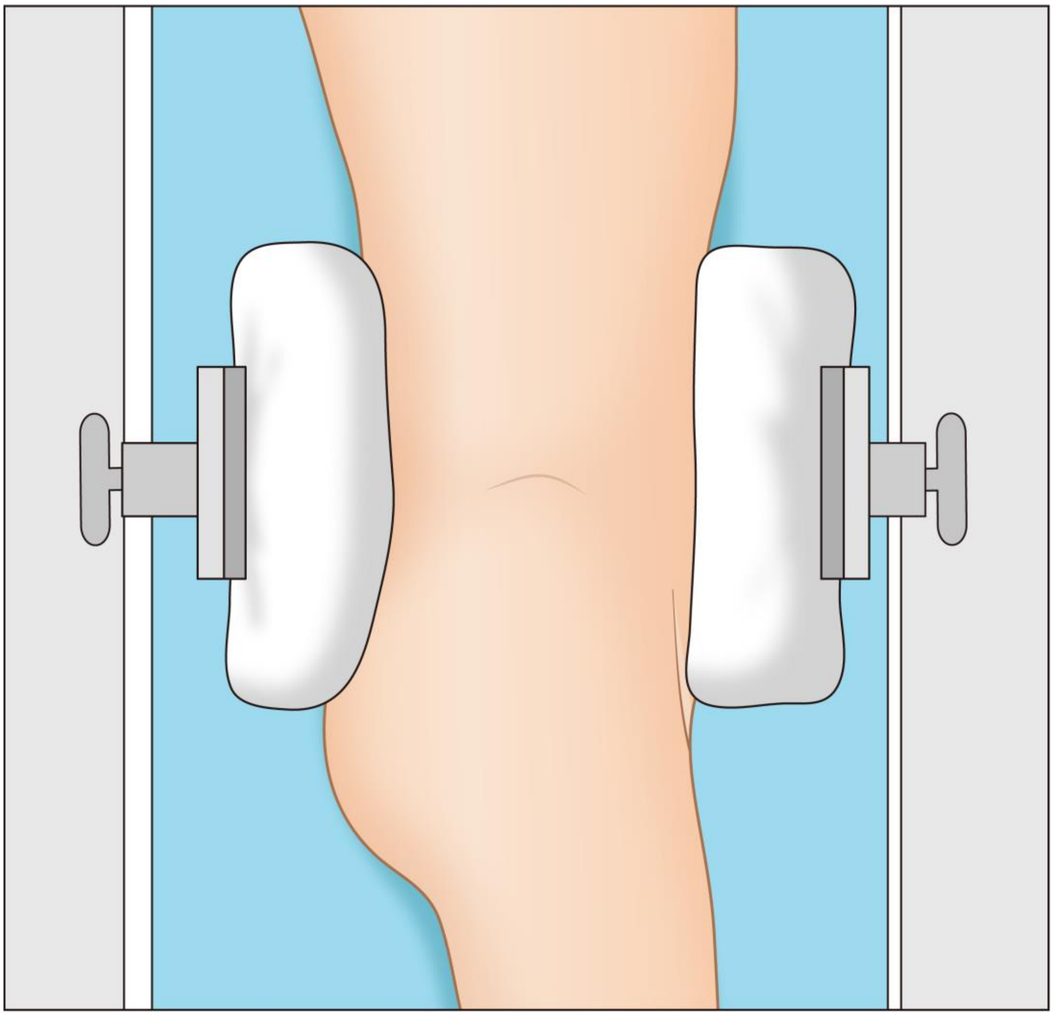

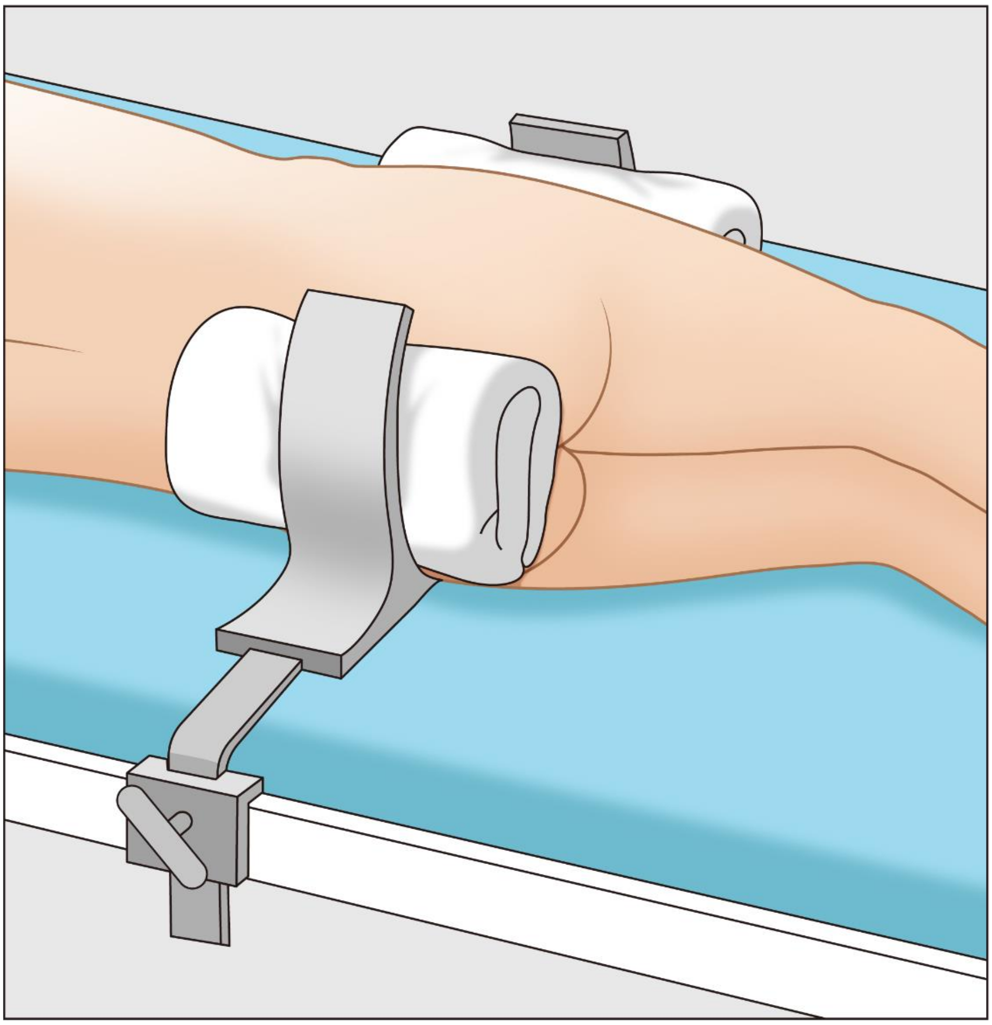

Supplement Figure 1. The lateral hip positioner secured the pelvis of the patient in lateral decubitus position.

Correspondence to: Jeong Joon Yoo, MD, PhD

Department of Orthopaedic Surgery, Seoul National University College of Medicine, Seoul, South Korea

101, Daehak-ro, Jongno-gu, Seoul, Republic of Korea

TEL: +82-2-2072-1994

FAX: +82-2-764-2718

E-mail: jjyos@snu.ac.kr
